# Integration of Two-Dimensional Liquid Chromatography-Mass Spectrometry and Molecular Docking to Characterize and Predict Polar Active Compounds in *Curcuma kwangsiensis*

**DOI:** 10.3390/molecules27227715

**Published:** 2022-11-09

**Authors:** Kaijing Xiang, Weijia Zhou, Tao Hou, Long Yu, Han Zhou, Liangliang Zhou, Yanfang Liu, Jixia Wang, Zhimou Guo, Xinmiao Liang

**Affiliations:** 1 CAS Key Laboratory of Separation Science for Analytical Chemistry, Dalian Institute of Chemical Physics, Chinese Academy of Sciences, Dalian 116023, China; 2 University of Chinese Academy of Sciences, Beijing 100049, China; 3 College of Fisheries and Life Science, Dalian Ocean University, Dalian 116023, China; 4 Jiangxi Provincial Key Laboratory for Pharmacodynamic Material Basis of Traditional Chinese Medicine, Ganjiang Chinese Medicine Innovation Center, Nanchang 330100, China

**Keywords:** *Curcuma kwangsiensis*, polar compounds, reversed-phase liquid chromatography × hydrophilic interaction chromatography, quadrupole time-of-flight mass spectrometry, molecular docking

## Abstract

*Curcuma kwangsiensis*, one species of *Curcumae zedoaria Ros. c*, is a commonly used traditional Chinese medicine (TCM) for treating cardiovascular disease, cancer, asthma and inflammation. Polar compounds are abundant in water decoction, which would be responsible for critical pharmacological effects. However, current research on polar compounds in *Curcumae zedoaria Ros. c* remains scarce. In this study, the polar fraction from *Curcuma kwangsiensis* was firstly profiled on G protein-coupled receptor 109A (GPR109A), β2-adrenergic receptor (β2-AR), neurotensin receptor (NTSR), muscarinic-3 acetylcholine receptor (M3) and G protein-coupled receptor 35 (GPR35), which were involved in its clinical indications and exhibited excellent β2-AR and GPR109A receptor activities. Then, an offline two-dimensional reversed-phase liquid chromatography (RPLC) coupled with the hydrophilic interaction chromatography (HILIC) method was developed to separate polar compounds. By the combination of a polar-copolymerized XAqua C18 column and an amide-bonded XAmide column, an orthogonality of 47.6% was achieved. As a result of coupling with the mass spectrometry (MS), a four-dimensional data plot was presented in which 373 mass peaks were detected and 22 polar compounds tentatively identified, including the GPR109A agonist niacin. Finally, molecular docking of these 22 identified compounds to β2-AR, M3, GPR35 and GPR109A receptors was performed to predict potential active ingredients, and compound 9 was predicted to have a similar interaction to the β2-AR partial agonist salmeterol. These results were supplementary to the material basis of *Curcuma kwangsiensis* and facilitated the bioactivity research of polar compounds. The integration of RPLC×HILIC-MS and molecular docking can be a powerful tool for characterizing and predicting polar active components in TCM.

## 1. Introduction 

*Curcuma kwangsiensis* (*C. kwangsiensis*), one species of *Curcumae zedoaria Ros. c* (*C. zedoaria*), is a commonly used traditional Chinese medicine (TCM) belonging to the Zingiberaceae family [1]. It is a clinically used drug for promoting blood circulation and relieving pain [2]. Currently, research on the chemical constituents of *C. zedoaria* is mainly concentrated on sesquiterpenes, diterpenoids and diarylheptanoids [3,4,5,6], which are related to antitumor, anti-inflammatory and anti-asthmatic effects [7,8,9]. These active compounds are mainly medium polar or weakly polar components. Considering the clinical use of *C. zedoaria* is generally taken with water decoctions, polar compounds are also important active ingredients. Few reports focused on polar ingredients in *C. kwangsiensis* [10]. Therefore, the investigation of polar components in *C. kwangsiensis* is beneficial for elucidating their bioactivities.

The separation of polar compounds from TCM has remained challenging due to the poor retention on traditional reversed-phase liquid chromatography (RPLC) columns [11]. The polar-modified C18 stationary phase provides an alternative approach to solve this problem [12,13]; it contains polar-embedded, polar-endcapped and polar-copolymerized approaches [12,13,14]. As the RPLC surface bonded with polar groups, the retention of polar compounds is significantly enhanced, which also brings unique selectivity [15]. Further, due to the insufficient resolving power and peak capacity, it is difficult to achieve the comprehensive separation of polar components in *C. kwangsiensis* with one-dimensional liquid chromatography (1D-LC). Thus, it is necessary to develop a two-dimensional liquid chromatography (2D-LC) system by combining two orthogonal separation modes. Due to the compatibility issue of the mobile phase between the two dimensions in the online mode, the offline mode is easier to operate [16]. Hydrophilic interaction chromatography (HILIC), with a different retention mechanism from RPLC, served as an effective technique for separating polar compounds [15,17]. 

In the present study, we aim to separate and characterize polar components in *C. kwangsiensis.* The target activities of the polar fraction were first profiled; then, a 2D RPLC×HILIC system was constructed based on a polar-copolymerized XAqua C18 column and an XAmide column. Finally, the identified compounds in the polar fraction were subjected to docking-based virtual screening of four targets to predict active compounds.

## 2. Results

### 2.1. The Target Activity Profiling of Polar Fraction in C. kwangsiensis

A preparative Unitary C18 column was first employed to acquire the polar component of *C. kwangsiensis*, which is shown in Figure S1 (Supplementary Materials). The polar fraction was collected at a nearly dead time ranging from 3 min to 6 min. 

**Figure 1:**
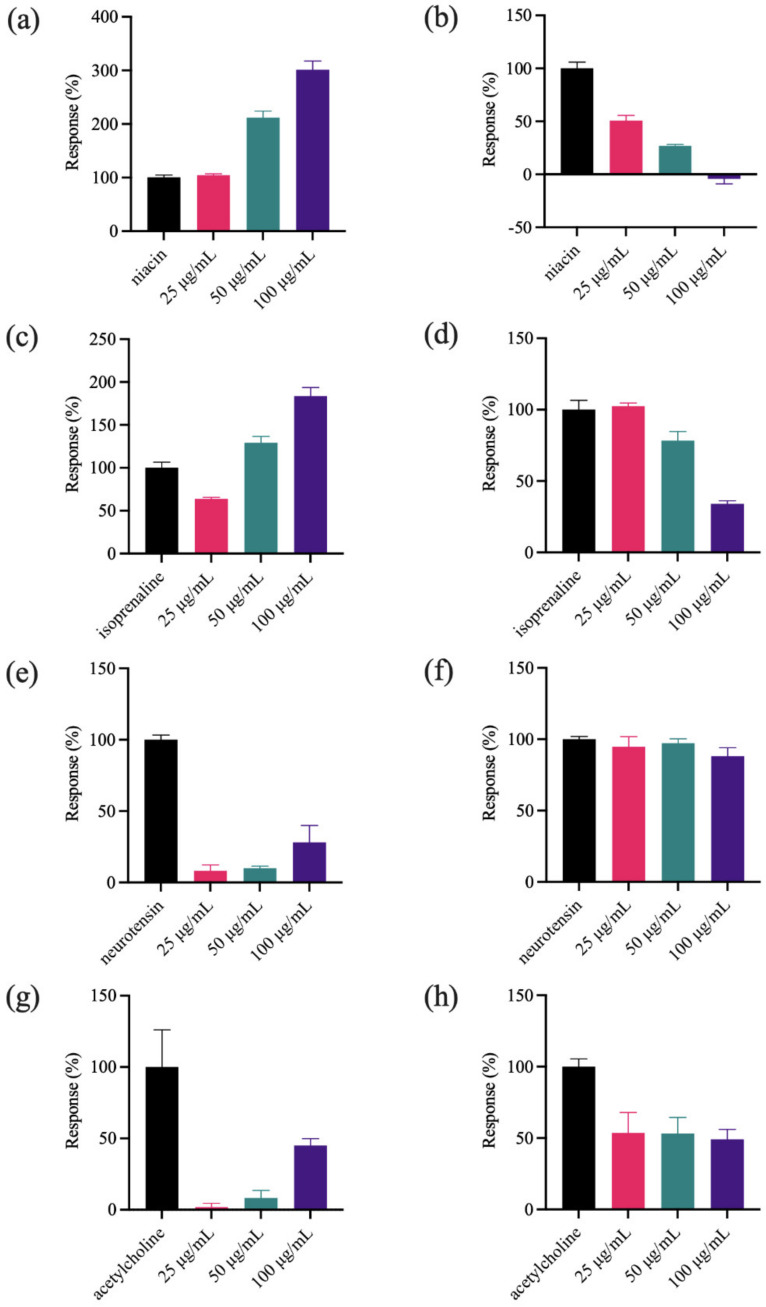

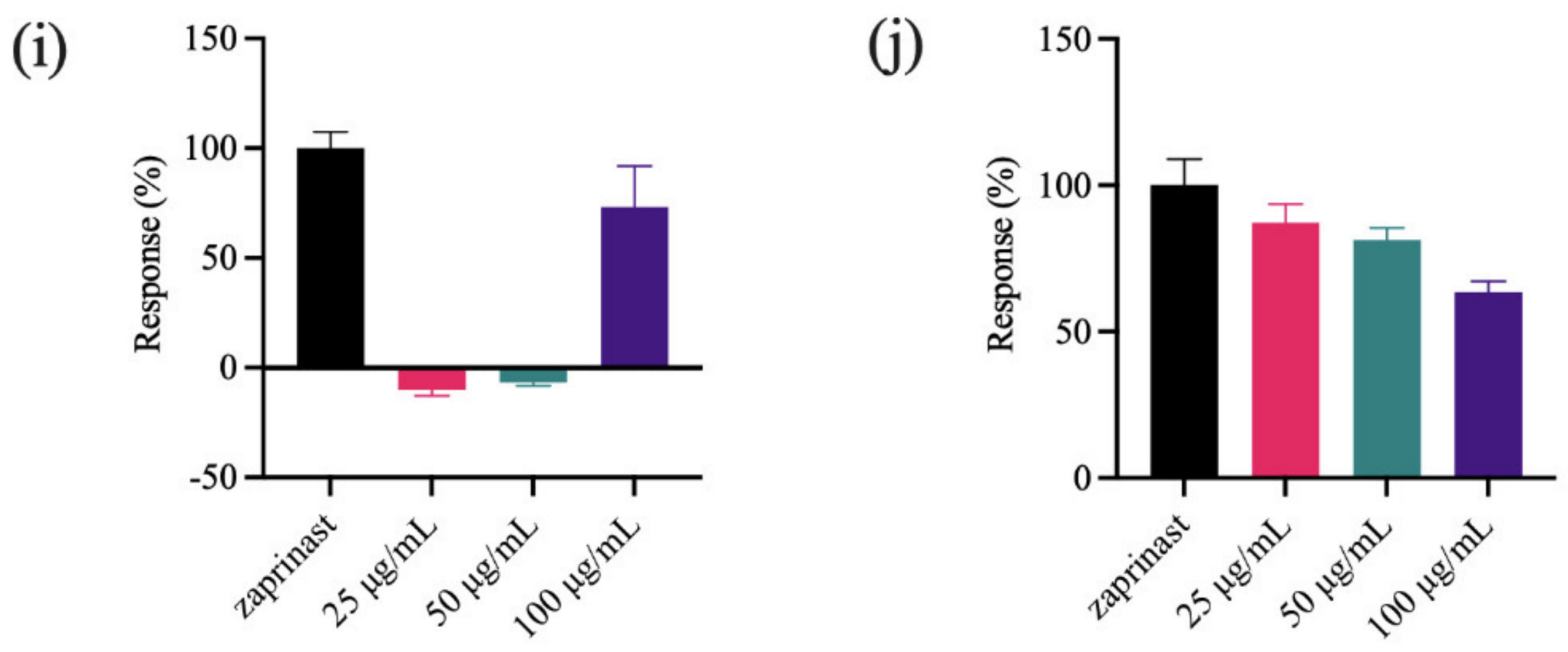
Target activity profiling of the polar fraction in (**a**−**d**) A431 and (**e**−**j**) HT-29 cells. (**a**,**c**,**e**,**g**,**i**) The DMR maximum amplitudes at post-stimulation of the polar fraction. The DMR maximum response of (**b**) niacin, (**d**) isoprenaline, (**f**) neurotensin, (**h**) acetylcholine and (**j**) zaprinast after the cells pre-stimulated with the polar fraction.

To identify the action targets of the polar ingredient, biological activity was profiled in related targets, including G protein-coupled receptor 109A (GPR109A), β2-adrenergic receptor (β2-AR), neurotensin receptor (NTSR), muscarinic-3 acetylcholine receptor (M3) and G protein-coupled receptor 35 (GPR35). Since A431 cells endogenously express GPR109A and β2-AR, and HT-29 cells express NTSR, M3 and GPR35, a two-step dynamic mass redistribution (DMR) assay was performed in A431 and HT-29 cells. The first step is to detect the DMR signals of the polar fraction on A431 cells (Figure 1a,c) and HT-29 cells (Figure 1e,g,i). The second step is to detect the DMR signals of the probe molecules pretreated with the polar fraction to elucidate the effects on GPR109A (Figure 1b), β2-AR (Figure 1d), NTSR (Figure 1f), M3 (Figure 1h) and GPR35 (Figure 1j). The fraction contained agonists if it triggered a significant DMR signal and reduced the receptor probe-induced DMR signal. In contrast, it contained antagonists if it had no DMR signal and blocked the receptor probe-induced DMR signal. Herein, the probe of GPR109A, β2-AR, NTSR, M3 and GPR35 was niacin, isoprenaline, neurotensin, acetylcholine and zaprinast, respectively. As shown in Figure 1a,c, the polar fraction showed concentration-dependent DMR signals in A431 cells, further attenuating the niacin-induced signals at all three concentrations (Figure 1b) and the isoprenaline-induced signal at 50 μg/mL and 100 μg/mL (Figure 1d), suggesting that the polar fraction may contain agonists of GPR109A and β2-AR receptors. As shown in Figure 1g,i, the polar fraction displayed weak DMR signals in HT-29 cells, further attenuated the acetylcholine-induced signal to approximately 50% (Figure 1h) and partially attenuated the zaprinast-induced signal in the second step (Figure 1j), indicating that the polar fraction may contain weak agonists of M3 and GPR35 receptors. The polar fraction had no significant effect on the neurotensin-induced signal (Figure 1e,f), suggesting it did not act on NTSR. The above profile analysis results indicated that the polar components in *C. kwangsiensis* possessed important bioactivity. Therefore, it is important to elucidate its material basis.

### 2.2. Construction of 2D-LC System for the Polar Fraction

**Figure 2:**
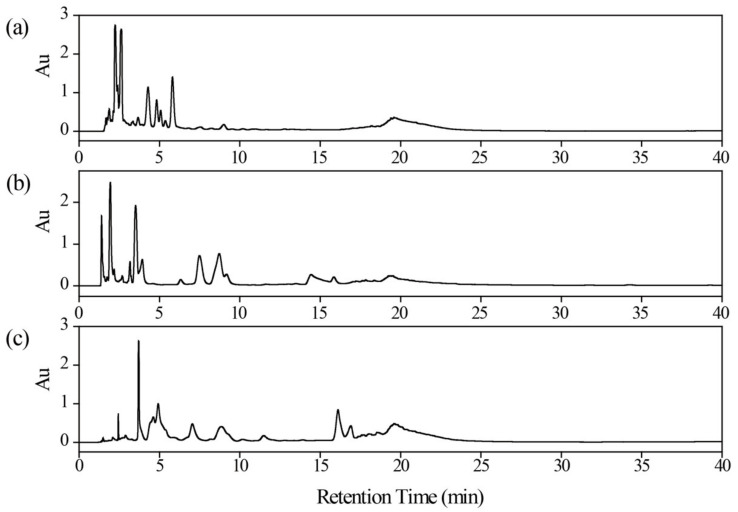
RPLC chromatograms of polar fraction at 254 nm on (**a**) XBridge column (150 × 4.6 mm i.d., 5 μm), (**b**) XCharge C18PN column (150 × 4.6 mm i.d., 5 μm) and (**c**) XAqua C18 column (150 mm × 4.6 mm i.d., 5 μm).

Considering the complexity of TCM and the limited resolving power of 1D-LC, an offline 2D-LC was designed to analyze the polar fraction to improve peak capacity and reduce sample complexity. Due to the poor solubility of polar components in organic solvent, RPLC was regarded as the first separation mode. Three RPLC columns bonded with different stationary phase groups were investigated, including XBridge C18 column, XCharge C18PN and XAqua C18. Figure 2 shows the separation of the polar fraction on these three columns. Polar constituents were eluted almost at the dead time on the XBridge C18 column, although they showed a well-shaped peak (Figure 2a). As shown on XCharge C18PN column (Figure 2b), the chromatographic peak density was reduced, and the retention of the latter peak was improved compared to XBridge C18, which may be related to the positive charge on the surface of the C18PN column. However, some polar compounds still exhibited poor retention on the XCharge C18PN column. The XAqua C18 column displayed better retention for polar components, evenly distributed in the separation chromatogram (Figure 2c). Eventually, the XAqua C18 column was chosen as the RPLC column. The mobile phase effect was also tested (Figure S2). The chromatograms showed no difference when using acetonitrile (ACN) or methanol (MeOH) as the organic phase. Due to the stronger elution ability of methanol in the next step of HILIC analysis, ACN was selected as the mobile phase for separating polar fractions on the XAqua C18 column.

**Figure 3:**
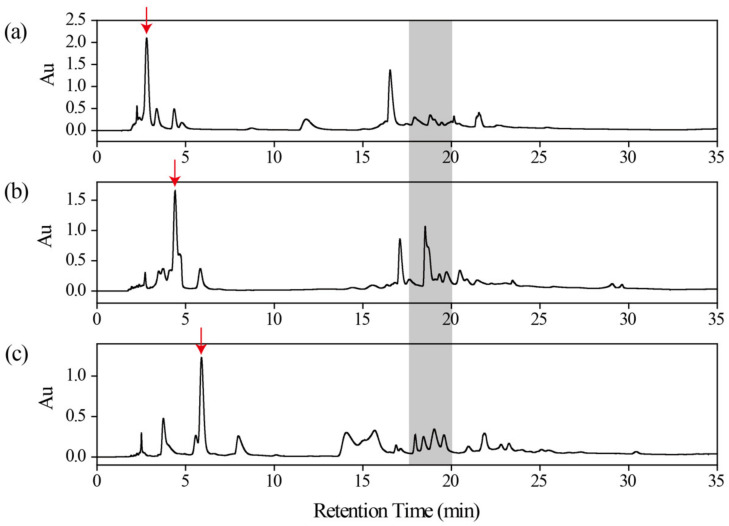
HILIC chromatograms of polar fraction at 254 nm on (**a**) BEH Silica column (150 × 4.6 mm i.d., 5 μm), (**b**) Inertsil Diol column (150 × 4.6 mm i.d., 5 μm) and (**c**) XAmide column (150 × 4.6 mm i.d., 5 μm). The red arrow indicated the polar compounds. The gray color indicated chromatographic peaks distributed on the above three columns.

In the case of HILIC separation, three HILIC columns were used to evaluate the retention for the polar fraction, including BEH Silica, Inertsil Diol and XAmide column, bonded with hybrid silica, diol and amide, respectively (Figure 3). It can be seen that polar compounds exhibited the strongest retention on XAmide column according to the main peak indicated by the arrow. And for the gray-shaded part, the chromatographic peaks were more widely distributed. Thus, the XAmide column was chosen as the HILIC column due to the strongest hydrophilic interaction and the most satisfactory separation ability. 

### 2.3. Offline 2D RPLC × HILIC System for Separating of Polar Fraction

**Figure 4:**
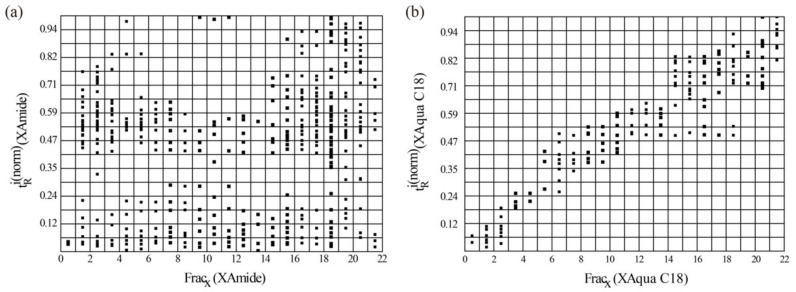
Normalized plots for 2D-LC systems: (**a**) XAqua C18 × XAmide system; (**b**) XAqua C18 × XAqua C18 system.

After optimizing the conditions of column and mobile phase, the RPLC separation and preparation of the polar fraction was conducted on a preparative XAqua C18 column. The resulting chromatogram is shown in Figure S3, which displayed a good separation for the polar fraction. A total of 22 fractions (F1–F22) were collected from 3 min to 25 min at 1 min interval. The 2D-LC separation of the polar fraction in *C. kwangsiensis* was shown in a three-dimensional projection chromatogram (Figure S4). More peaks were observed in 2D-LC compared to that in the 1D HPLC. Then, orthogonality was evaluated using the geometric approach. During the separation of these 22 fractions, 373 peaks were detected. According to Equation (2), the normalized data points were distributed in a space of 22 × 17 bins (374, which was approximate to 373) and bins containing data points were 182 (Figure 4a). $\Sigma \mathrm{bins}\;\left(\mathrm{blank}\right)$ was calculated by re-analyzing of the 22 fractions using the XAqua C18 column. As shown in Figure 4b, $\Sigma \mathrm{bins}\;\left(\mathrm{blank}\right)$ was 79. According to Equation (3), the orthogonality (O%) value was 43.7%. The results demonstrated that this 2D RPLC×HILIC system was highly orthogonal in the separation of polar components in *C. kwangsiensis*.

### 2.4. Analysis of Polar Compounds by Offline 2D RPLC × HILIC-Q-TOF-MS System

**Figure 5:**
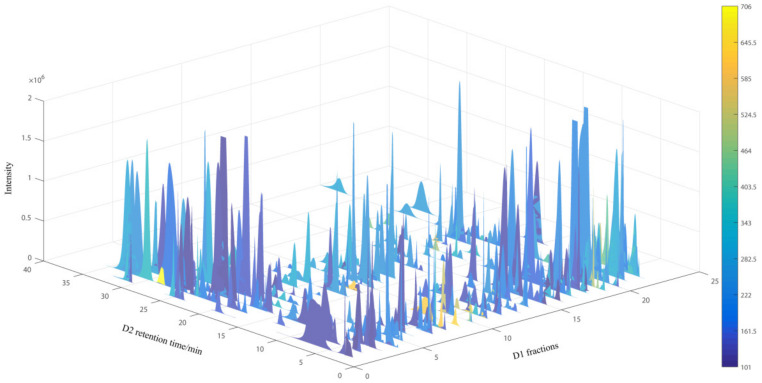
The four-dimensional plot of 2D RPLC×HILIC-Q-TOF-MS for the analysis of polar compounds in *C. kwangsiensis* (positive mode). Color bars represent the measured masses of components by Q-TOF-MS.

A quadrupole time-of flight (Q-TOF) MS instrument equipped with an ESI interface was connected with the 2D-LC system to analyze polar compounds in *C. kwangsiensis*. The high resolution of Q-TOF MS detection has proved indispensable to allow compound identification based on accurate molecular weights [18]. The mass data were collected in the positive mode, then processed by Agilent MassHunter software. The *m*/*z* values of [M + H]^+^, [M + Na]^+^, [M + K]^+^ or [M + NH_4_]^+^ for the components of each fraction were found. Then, we collected the raw EIC data of each component together with the exact mass, the first dimensional (D1) fraction number, and the second dimensional (D2) retention times of each peak. The processed data was later exported as a four-dimensional (4D) data plot (D1 fraction number and D2 retention time as x-axis and y-axis, peak intensity as z-axis, *m*/*z* as peak color) by MATLAB 6.5, illustrated in Figure 5. Each peak represented a compound, and 379 peaks were counted by the software. These peaks occupied a large portion of the 2D-LC space, which demonstrated a good separation of the polar components in *C. kwangsiensis*. The peak colors represented the mass range of the compounds and molecular weight distribution. Most polar compounds in *C. kwangsiensis* had a molecular weight below 500, and partial compounds with larger molecular weight (yellow color) were distributed in F8, F9 and F11, which would provide guidance for the novel compound discovery. These results illustrated the excellent separation ability and detection resolution of this off-line 2D LC-Q-TOF-MS system. Besides, this 4D chromatography plot provided narrower peak widths, so peak overlap was greatly reduced and components with lower concentration were obtained.

### 2.5. Characterization of Polar Compounds in C. kwangsiensis

Based on accurate molecular weights and fragmentation information obtained from simultaneous MS/MS acquisition in the positive mode, compound names would be tentatively speculated through the literature review. However, due to most reports being focused on the investigation of volatile ingredients or weak polar ingredients in *C. kwangsiensis*, only a total of 22 polar compounds were tentatively characterized from the polar fraction in *C. kwangsiensis*, which are shown in Table 1. 

### 2.6. Virtual Screening of Identified Compounds to Four Targets

**Figure 6:**
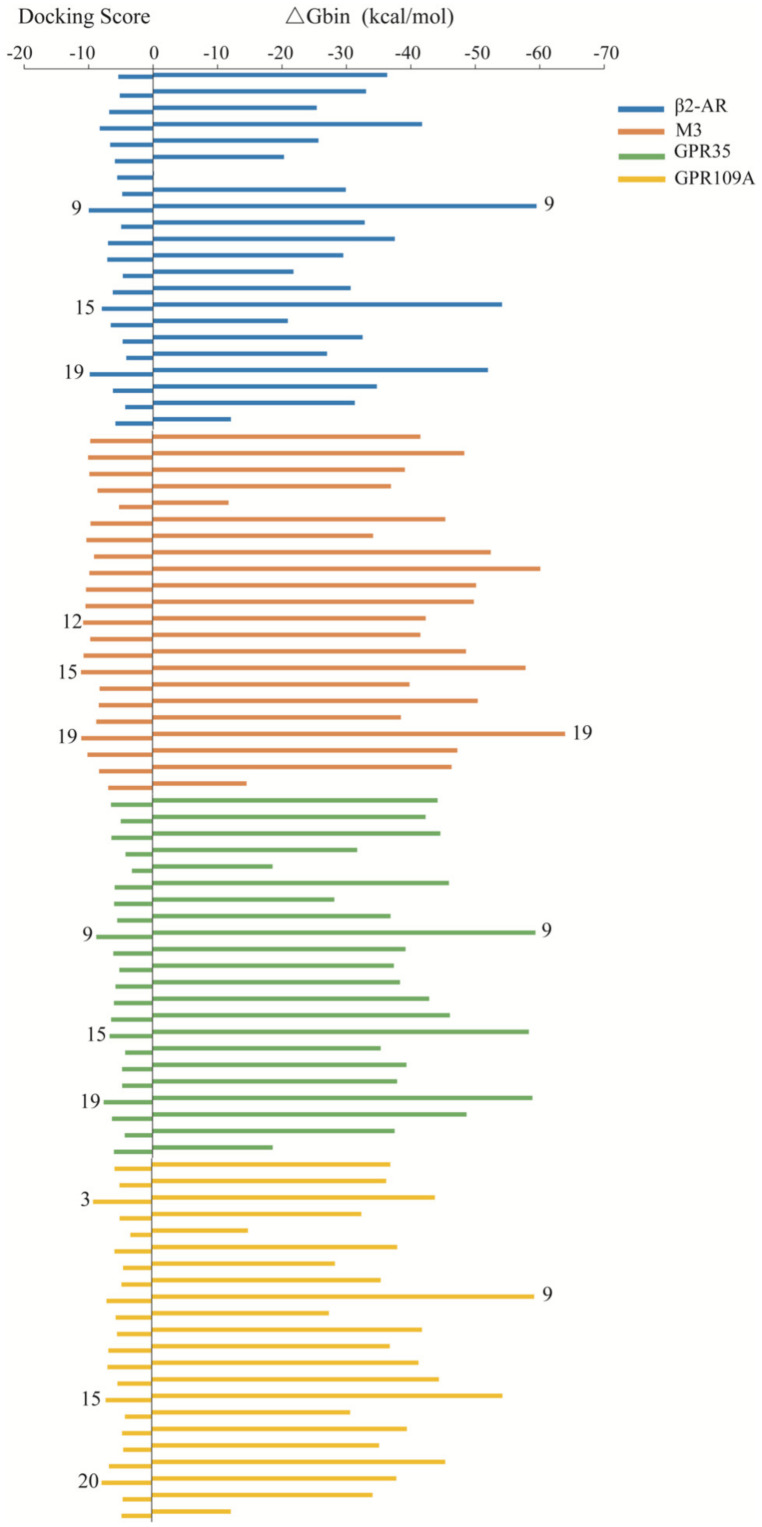
Docking scores and binding free energies of the 22 identified compounds to β2−AR, M3, GPR35 and GPR109A receptors.

In our above studies, we found that the polar fraction from *C. kwangsiensis* may contain agonists of GPR109A and β2-AR receptors, and weak agonists of M3 and GPR35 receptors. Therefore, after we identified 22 compounds in the polar fraction, in silico prediction of their binding potentials was conducted on the above targets. Docking scores and binding free energies of the compounds binding with β2-AR, M3, GPR35 and GPR109A are shown in Figure 6. For each receptor, ligands with the top three docking scores were as follows: for β2-AR, compound 9 (−9.98), compound 15 (−7.95), compound 19 (−9.83); for M3, compound 12 (−10.54), compound 15 (−10.87), compound 19 (−10.83); for GPR35, compound 9 (−8.54), compound 15 (−6.53), compound 19 (−7.42) and for GPR109A, compound 3 (−7.93), compound 15 (−6.23), compound 20 (−6.77). A very negative docking score suggests the potential for strong binding of a ligand to the target receptor, which means that the ligand has a potential target activity. Considering their binding free energy, the highest potential compounds were compound 19 for M3 and compound 9 for β2-AR, GPR35 and GPR109A.

### 2.7. Molecular Docking and Dynamics Simulation

**Figure 7:**
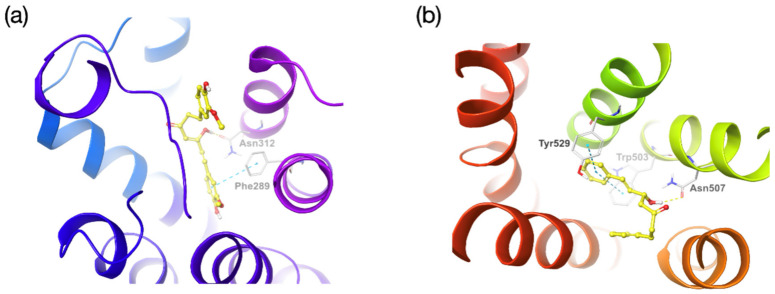
Docking poses of (**a**) compound 9 to β2-AR and (**b**) compound 19 to M3. The ligands and amino acid residues of the binding pockets are shown in the sticks. The hydrogen and pi-pi bonds are represented as yellow and blue dashed lines, respectively. The carbon atoms of the ligands and the residues are colored yellow and grey, respectively. Nitrogen, oxygen and hydrogen atoms are colored blue, red and white, respectively.

Given that the crystal structure of β2-AR (human) and M3 (rat) are known, molecular docking was conducted on compound 9 and compound 19 with corresponding receptors. Compound 9 had binding interactions with β2-AR via hydrogen bond with the key amino acid residue Asn312 and pi-pi interaction with Phe289 (Figure 7a). In the M3-compound 19 docking model, pi-pi interactions were observed at key residues Trp503 and Tyr529 and hydrogen bonding was observed at Asn507 (Figure 7b). 

We performed a molecular dynamic (MD) simulation about the β2-AR-compound 9 complex to claim the time-dependent stability of protein-ligand interactions. Root-mean-square deviation (RMSD) for the protein-ligand complex is shown in Figure S5a; the curve exhibited a fluctuation within 3.0 Å, which demonstrated the stability of the ligand within the binding pocket. Further, the root mean square fluctuation (RMSF) plot of the complex revealed that most residues fluctuated below 3.0 Å; few residues showed fluctuation up to 5.0 Å, and those with ligand interactions fluctuated below 1.5 Å (Figure S5b). The H-bonding interactions were formed with Asp113, Ser203, Ser207, Phe193 and Asn312, which contributed to the binding affinity of the ligand (Figure S5c). 

## 3. Discussion

*Curcuma kwangsiensis* is considered as a clinically used drug for various diseases. Research mainly concentrated on components such as sesquiterpenes, diterpenoids and diarylheptanoids, while the active components and main action targets were not fully researched. In this study, we focused on the rarely reported polar compounds that were challenging to separate due to their poor retention. Firstly, the biological activity of the polar ingredient was profiled in related targets according to its clinical indications, including GPR109A (cardiovascular disease), β2-AR (asthma), NTSR (cancer), M3 (asthma) and GPR35 (inflammation). By a two-step DMR assay, concentration-dependent activation signals were observed in GPR109A and β2-AR receptors, suggesting that the polar fraction may contain GPR109A and β2-AR receptor agonists. 

Based on the important bioactivity, we developed an offline 2D RPLC × HILIC-Q-TOF-MS/MS system to separate and analyze polar components. With the introduction of polar groups to the stationary phase, the XAqua C18 column displayed better retention for polar components. Moreover, the XAqua C18 column could be used under a 100% aqueous mobile phase, suggesting its great potential as the first dimensional RPLC column. For the second dimensional HILIC system, the amide-bonded XAmide column was chosen to maximize retention time compared with the hybrid silica and diol-bonded column. As a result of its quite different retention mechanisms, the offline 2D RPLC × HILIC-Q-TOF-MS/MS achieved a high separation orthogonality value of 43.7%, and 22 polar compounds were tentatively characterized. They were assigned to 13 sesquiterpenes, 3 diarylheptanoids, 2 organic acids, 2 alkaloids and 2 other compounds. Among them, compound 5 was identified as niacin, an agonist of the GPR109A receptor. 

Molecular docking-based virtual screening techniques are essential for studying the interactions of ligands and proteins. We selected 22 compounds to analyze their binding potentials on the above targets. Compound 9 showed the lowest binding free energies on the β2-AR, GPR35 and GPR109A receptors, and compound 19 showed the lowest binding free energy on M3, suggesting the most significant possibility of occurrence of the binding reaction. Given that the crystal structure of human β2-AR was known, we performed MD to illustrate the stability of compound 9 into the active site of β2-AR. Interestingly, the key amino acids that form hydrogen bonds with protein residues highly overlapped with those of salmeterol (Asp113, Ser203, Phe193, Asn312) [26], indicating that compound 9 had a similar interaction to the β2-AR partial agonist, salmeterol. Our findings expanded the polar ingredients and their potential targets of *C. kwangsiensis*. 

## 4. Materials and Methods

### 4.1. Chemicals and Instruments

HPLC-grade ACN and MeOH were purchased from Yuwang Chemical Reagent Factory (Shandong, China). HPLC-grade formic acid (FA) was from J&K Chemical (Hebei, China). Isoprenaline and acetylcholine were purchased from Amquar Biological Technology Co., Ltd. (Shanghai, China), Niacin, neurotensin and zaprinast were bought from energy chemical (Shanghai, China), Macklin Biochemical Co., Ltd. (Shanghai, China) and Sigma Chemical Co. (St. Louis, MO, USA), respectively. Ultrapure water (H_2_O) was filtered by a Milli-Q purification system from Millipore (Billerica, MA, USA). The dried rhizomes of *C. kwangsiensis* were from Nanning, Guangxi Province (China), identified by Dr. Xiaoping Yang, Dalian Institute of Chemical Physics, Chinese Academy of Sciences. The HPLC system (Waters, Milford, MA, USA) consisted of an Alliance 2695 quaternary gradient pump, an autosampler, a column thermostat system, and a UV detector. A 2489 UV detector (Waters) was used for sample preparation, and a 2998 UV detector (Waters) was used for analytical work. The Empower 3.0 workstation software was used to manipulate the HPLC system and acquire the chromatographic data. The MS determination was performed on an Agilent 6540 UHD Q-TOF with electrospray ionization ion source in positive ion mode. Mass hunter workstation software Qualitative Analysis B.04.00 was used for processing MS data. 

The columns used in this work consisted of Unitary C18 column (265 mm × 50 mm i.d., 7 μm, Acchrom, Beijing, China), XAqua C18 column (250 mm × 10 mm i.d., 5 μm, Acchrom), XAqua C18 column (150 mm × 4.6 mm i.d., 5 μm, Acchrom), XCharge C18PN column (150 × 4.6 mm i.d., 5 μm, Acchrom), XBridge column (150 × 4.6 mm i.d., 5 μm, Acchrom), XAmide column (150 × 4.6 mm i.d., 5 μm, Acchrom), Inertsil Diol column (150 × 4.6 mm i.d., 5 μm, GL Sciences), BEH Silica column (150 × 4.6 mm i.d., 5 μm, Acchrom) and XAmide column (150 × 2.1 mm i.d., 5 μm, Acchrom).

### 4.2. Cell Culture

Human colon cancer cell line HT-29 and human epidermoid carcinoma cell line A431 were purchased from the Cell Bank of Type Culture Collection of Chinese Academy of Sciences (CAS, Shanghai, China). HT-29 was cultured in McCoy’s 5A Medium with 10% FBS, 100 μg/mL penicillin and 100 μg/mL streptomycin. A431 was cultured in Dulbecco’s Modified Eagle’s Medium (DMEM) with a mixture of 10% FBS, 4.5 g L/glucose, 2 mM glutamine, 100 μg/mL penicillin and 100 μg/mL streptomycin. Cells were cultured in a 37°C humidified incubator with 5% CO_2_. 

### 4.3. Sample Preparation

Five kilograms of *C. kwangsiensis* tuber were sliced into pieces and refluxed in 70% ethanol (50 L) for three times, 2 h each. The solution was then condensed to the volume of 5.7 L using a rotary evaporator in a high vacuum at 60 °C. The concentrated solution was then partitioned successively with n-heptane (3 × 5.7 L) and ethyl acetate (5 × 5.7 L). The resulting ethyl acetate solution was further dried under vacuum and re-dissolved in 50% MeOH/H_2_O (*v*/*v*) with a 200 mg/mL concentration. The supernatant was centrifuged at 10000 r/min for 5 min and filtered through a 0.45 μm membrane.

The polar fraction preparation was performed on the Purification Factory (Waters, Milford, MA, USA) consisting of two 2525 binary gradient modules (Waters, Milford, MA, USA), an autosampler (Leap Technologies, Carrboro, NC, USA) and a 2498 UV detector (Waters), using a Unitary C18 column (265 mm × 50 mm i.d., 7 μm, Acchrom). Mobile phase A was 0.1% FA (*v*/*v*) in aqueous solution, and B was MeOH. The gradient elution conditions were set as follows: 0 min (25% B)—5 min (30% B)—15 min (45% B)—40 min (70% B)—50 min (95% B)—60 min (95% B). The flow rate was 80 mL/min. The chromatogram was recorded at 254 nm, and the polar fraction was collected from 3 to 6 min. 

### 4.4. Chromatographic Conditions

In the 2D RPLC × HILIC analysis, the first-dimensional separation was performed on XAqua C18 column (150 mm × 4.6 mm i.d., 5 μm), XCharge C18PN column (150 × 4.6 mm i.d., 5 μm) and XBridge column (150 × 4.6 mm i.d., 5 μm). The corresponding mobile phase A was ACN with 0.1% FA (*v*/*v*), and the mobile phase B was H_2_O with 0.1% FA (*v*/*v*). The linear gradient was 0 min (0%A)—6 min (0%A)—13 min (2%A)—16 min (8%A)—17 min (14%A)—20 min (24%A)—30 min (40%A)—35 min (70%A)—40 min (95%A)—45 min (95%A). The second-dimensional analysis was performed on an XAmide column (150 × 4.6 mm i.d., 5 μm, Acchrom), Inertsil Diol column (150 × 4.6 mm i.d., 5 μm) and BEH Silica column (150 × 4.6 mm i.d., 5 μm). The linear gradient was as follows: 0 min (98%A)—12 min (98%A)—13 min (92%A)—30 min (75%A)—40 min (50%A)—50 min (5%A)—55 min (5%A). The flow rate was 1.0 mL/min.

RPLC preparation was performed on XAqua C18 column (250 mm × 10 mm i.d., 5 μm), the gradient elution was: 0 min (0%A)—6 min (0%A)—13 min (2%A)—16 min (8%A)—17 min (14%A)—20 min (24%A)—30 min (40%A)—35 min (70%A)—40 min (95%A)—45 min (95%A) at a flow rate of 3.3 mL/min. Fractions were collected manually from 3 to 25 min at 1 min intervals and labeled as F1–F22 in order. The fractions were evaporated to dryness under a nitrogen stream, and the residue was dissolved in ACN/H_2_O (3:1, *v*/*v*) for the second-dimensional separation. The injection volume was 100 μL for the first dimension and 10 μL for the second dimension, respectively. In both systems, the column temperature was maintained at 30 ℃, and the UV detection was set at 254 nm.

### 4.5. Mass Spectrometry Analysis

An Agilent 6540 UHD Accurate Q-TOF mass spectrometer (Agilent, Palo Alto, Santa Clara, CA, USA) was coupled to the offline 2D RPLC × HILIC system. The ion source was operated in the positive ion mode. High-purity nitrogen (N_2_) was used as sheath gas (8 L/min) and nebulizing gas (35 psi). Other parameters were as follows: capillary voltage, 3500 V; collision energy, 25 eV; fragmentor voltage, 75 V; skimmer voltage, 65 V; octopole 1 rf voltage, 750 V; data acquisition, 1 spectrum/s. The MS scan ranged from 100 to 1000 *m*/*z*. The data were processed using MassLynx 4.1 software.

### 4.6. Dynamic Mass Redistribution Assay

DMR assays were carried out using an Epic^®®^ BT system (Corning, NY, USA). HT-29 and A431 cells were directly seeded in Epic 384-well biosensor microplates with a density of 30,000 per well and 20,000 per well, respectively. HT-29 cells were cultured in the complete medium overnight. A431 cells were cultured in the complete medium overnight, followed by overnight starvation in the serum-free medium. Once the cells formed a monolayer with a confluence of ~95%, they were manually washed and maintained in 30 μL HBSS buffer for 1 h in the Epic system. The polar fraction was dried under vacuum, re-dissolved in dimethyl sulfoxide (DMSO) to 100 mg/mL and diluted with HBSS buffer before analysis.

For profiling the polar fraction, a two min baseline was first established, followed by adding polar fraction (25 μg/mL, 50 μg/mL and 100 μg/mL) and monitoring the fraction-induced DMR signals for 1 h. Then, a 2 min baseline was re-established, followed by adding niacin (10 μM) and isoprenaline (1.25 nM) in A431 cells for GPR109A receptor and β2-AR, neurotensin (4 nM), acetylcholine (10 μM) and zaprinast (2.5 μM) in HT-29 cells for neurotensin receptor (NTSR), muscarinic M3 receptor and GPR35 receptor, respectively. Then, the DMR signals were monitored for another 1 h. 

### 4.7. Virtual Screening and Molecular Docking

The crystal structures of the β2-AR receptor (PDB entry: 6MXT) and M3 receptor (PDB entry: 4DAJ) were used as templates in docking-based virtual screening [26,27]. Molecular Docking was conducted in the ‘Glide docking’ module of Schrödinger Suite 2022-01 modeling software suite. Using the Prime module, hydrogen atoms were added, and crystal water was deleted in the crystal structures. Further, partial charges were distributed using the ‘Optimized Potentials for Liquid Simulations-4’ (OPLS-4) force field, and the structures were minimized until RMSD attained the maximum value of 0.3 Å. Minimized 3D structures of the 22 identified ligands were generated using the OPLS-4 force field in the LigPrep module. For the M3 complex, a docking pocket of a size of 10 Å × 10 Å × 10 Å was generated after the removal of the ligand that was initially co-crystalized with the receptor in the complex using the ‘Receptor Grid Generation’ module. For the β2-AR complex, a docking pocket of a size of 15 Å × 14 Å × 16 Å was generated similarly.

Since 3D protein structures of the GPR35 and GPR109A receptors have not been determined, we used protein structures predicted by AlphaFold2 (GPR35: AF-Q9HC97-F1 and GPR109A: AF-Q8TDS4-F1) in corresponding calculations. For the predicted protein structure of GPR35, we applied CavityPlus Server to detect the binding pocket position and key residues [28,29,30]. A grid box for docking (10 Å × 10 Å × 10 Å) was generated based on the center of the key residues around the detected binding pocket using the ‘Receptor Grid Generation’ of the Schrödinger software. For the AlphaFold2-predicted GPR109A, a binding pocket was predicted by the ‘Site Map’ module of the Schrödinger software. Then, Induced Fit Docking (IFD) was performed on the predicted structure and a known GPR109A-active compound SCH-900271 (PubChem CID 56950369). After evaluation of the generated docking poses, an optimal pose was selected, and a binding pocket (14 Å × 14 Å × 14 Å) was generated based on this pose by the ‘Receptor Grid Generation module’. 

After we defined the binding pockets of the four receptors, the docking-based virtual screening was separately performed using the ‘Extra precision’ (XP) mode of the Glide program. Calculation of binding free energy (∆G_bin_) for generated docking poses was achieved through the Molecular Mechanics-Generalized Born Surface Area (MM-GBSA) approach.

### 4.8. Molecular Dynamics Simulation

MD simulations were performed with the docking complex of β2-AR (PDB:6MXT) with compound 9 by using Desmond of Schrödinger 2022-01 modeling software [31] suite with 15 ns simulation time. Periodic boundary conditions were constructed using a DPPC lipid membrane and a well-established SPC water model with orthorhombic intermittent limit conditions for a 10 Ã buffer region. The OPLS4 molecular mechanic’s force field was used for performing the initial steps. Na^+^ was added to neutralize the entire framework of atoms. The trajectory was kept at 5.0 ps time and the number of frames at 3000. The Ensemble class was kept at NPγT with a temperature of 300 K and pressure at 1.01325 bar. 

### 4.9. Data Analysis

The orthogonality of selected 2D-LC modes was calculated using a geometric approach [32]. Retention data were acquired in a single-dimensional LC setup for each LC mode and normalized according to Equation (1).

(1) $${\mathrm{RT}}_{i\left(\mathrm{norm}\right)}=\frac{{\mathrm{RT}}_{i}-{\mathrm{RT}}_{\min}}{{\mathrm{RT}}_{\max}-{\mathrm{RT}}_{\min}}$$

RT_max_ and RT_min_ represent the retention times of the most and least peaks in the data set. The retention times RT_i_ are converted to normalized RT_i(norm),_ whose values range from 0 to 1.

In the 2D-LC separation space, for the x-axis, the grid number is equal to the first-dimensional fractions. For the y-axis, the grid number is calculated according to Equation (2).

(2) $${\mathrm{Grid}}_{y}=\frac{{\mathrm{Num}}_{p}}{{\mathrm{Frac}}_{x}}$$

Num_p_ represents the number of peaks detected by 2D-LC, and Frac_x_ is the number of the first-dimensional fractions. The calculation of the orthogonality is performed according to Equation (3).

(3) $$O\%=\frac{\Sigma \mathrm{bins}-\Sigma \mathrm{bins}\;\left(\mathrm{blank}\right)}{0.63P_{\max}}\times 100\%$$

Σbins is the number of bins that contain data points in the 2D-LC separation space. P_max_ is the sum of bins represented total peak capability. $\Sigma \mathrm{bins}\;\left(\mathrm{blank}\right)$ is the number of grids using the same conditions as the first dimension.

All DMR data were obtained using Epic Imager software (Corning, NY, USA) and processed with Imager Beta 3.7 (Corning), Microsoft Excel 2016 and GraphPad Prism 6 (GraphPad Software Inc., San Diego, CA, USA). The signals of each DMR with the maximum response were extracted and used for analysis. All DMR signals were background-deducted. 

## 5. Conclusions

In this study, we investigated the polar components in *C. kwangsiensis* and their potential targets using integration of two-dimensional liquid chromatography-mass spectrometry and molecular docking. To elucidate the basis of its components, an offline 2D-LC system was set up by combining a polar-copolymerized XAqua C18 column and an amide-bonded XAmide column. The combination solved the problem of poor retention of polar ingredients and exhibited good separation selectivity and orthogonality. Finally, 379 mass peaks were detected, and 22 polar compounds were grouped and tentatively identified in *C. kwangsiensis*, including the GPR109A agonist, niacin. The identified compounds were also subjected to docking-based virtual screening of four targets related to clinical indications of *C. kwangsiensis*. MD on β2-AR-compound 9 complex indicated that compound 9 had a similar interaction to the β2-AR partial agonist, salmeterol. Our results provided an effective supplement for the material basis study of *C. kwangsiensis* and provided guidance for the bioactivity research of polar compounds. The developed method also provided an efficient tool for researching polar compounds in TCM.

## Figures and Tables

**Table 1:** Tentatively identified chemical constituents of *C. Kwangsinesis* by comprehensive 2D LC-Q-TOF MS/MS.

No.	1D Fraction	^2^Dt_R_/min	Formula	*m*/*z*	Measured Ions	Mass Error/ppm	Fragments	Tentative Identification	Reference or Database
1	F1	25.331	C15H22O3	251.1616	[C15H23O3]^+^	10.28	235.1309, 189.0848, 175.1196,159.0911,145.1122	aerugidiol	[19]
2	F2	32.759	C15H18O3	269.1167	[C15H18O3Na]^+^	−7.66	205.0979, 137.0462, 61.0285	zedoarol	[20]
3	F3	2.83	C11H19NO6	262.1296	[C11H20NO6]^+^	−4.01	216.1239, 161.0686, 118.0867, 72.0812	heterodendrin	[21]
4	F3	9.872	C8H11NO	138.0912	[C8H12NO]^+^	0.75	122.0594, 108.0806, 91.0536	tyramine	[20]
5	F3	30.911	C6H5NO2	124.0383	[C6H6NO2]^+^	8.17	80.0488, 106.0276	niacin	[22]
6	F4	21.339	C15H26O2	261.1837	[C15H26O2Na]^+^	−5.03	199.0450, 149.0239, 90.9767	polydactin B	[23]
7	F5	17.259	C19H30O	313.2102	[C19H30ONa]^+^	12.41	247.0530, 129.0516, 113.0594	curcuminol E	[21]
8	F6	16.918	C15H18O	215.1399	[C15H19O]^+^	14.67	136.0620, 99.0554, 70.0655	8-hydroxycadalene	[24]
9	F7	1.012	C21H22O6	371.1455	[C21H23O6]^+^	9.23	327.1731, 311.1783, 149.0247	dihydrocurcumin	[19]
10	F7	2.701	C15H24O3	275.1627	[C15H24O3Na]^+^	−3.7	201.0449, 129.0516	zedoarondiol	[20]
11	F7	2.701	C15H22O2	235.1698	[C15H23O2]^+^	−2.22	118.0863	curcumenone	[21]
12	F7	21.414	C15H20O4	287.1254	[C15H20O4Na]^+^	6.74	191.0802, 163.0858, 61.0282	curcumenolactone C	[21]
13	F7	21.494	C15H20O4	287.1340	[C15H20O4Na]^+^	5.23	165.0676, 137.0701	curcolonol	[21]
14	F7	25.652	C15H22O5	305.1367	[C15H22O5Na]^+^	−2.68	227.1027, 191.0817, 149.0712	zedoarolide B	[20]
15	F8	20.693	C19H20O	265.1588	[C19H21O]^+^	−0.41	195.1135, 136.0618, 70.0654	trans-1, 7-diphenyl-1, 3-heptadien-5-ol	[21]
16	F15	5.743	C10H12O	171.0762	[C10H12ONa]^+^	12.4	120.0465, 86.0595	cuminaldehyde	[9]
17	F16	19.487	C15H16O2	251.1017	[C15H16O2Na]^+^	11.18	95.0610	gweicurculactone	[20]
18	F18	18.378	C15H20O3	271.1301	[C15H20O3Na]^+^	1.47	173.0825, 101.0247, 730.659	curdionolide B	[20]
19	F18	21.977	C19H20O2	281.1515	[C19H21O2]^+^	7.52	265.1285	(*E*)-1,7-diphenyl-3-hydroxy-1-hepten-5-one	[20]
20	F20	10.539	C15H20O5	281.1384	[C15H21O5]^+^	−0.18	165.0557, 123.0801, 89.0610	zedoalactone B	[20]
21	F20	16.537	C15H18O2	231.135	[C15H19O2]^+^	12.85	175.1236, 137.0591	epicurzerenone	[25]
22	F22	15.6	C5H7NO3	130.0511	[C5H8NO3]^+^	−9.49	56.0502, 84.0453, 119.0362	pyroglutamic acid	[20]

## Data Availability

The data supporting this study’s findings are available upon request.

## Supplementary Materials

The following supporting information can be downloaded at: https://www.mdpi.com/article/10.3390/molecules27227715/s1, Figure S1. HPLC preparation of polar fraction on the Unitary C18 column (265 mm × 50 mm i.d., 7 μm); Figure S2. RPLC chromatograms of polar fraction at 254 nm under (a) mobile phase A: 0.1% formic acid in water (*v*/*v*), mobile phase B: 0.1% formic acid in MeOH (*v*/*v*), (b) mobile phase A: 0.1% formic acid in water (*v*/*v*), mobile phase B: 0.1% formic acid in ACN (*v*/*v*); Figure S3. The first-dimension separation of the polar fraction at 254 nm on the XAqua C18 column (250 mm × 10 mm i.d., 5 μm); Figure S4. The three-dimensional chromatogram of 22 fractions at 254 nm analyzed on the XAmide column (150 × 2.1 mm i.d., 5 μm); Figure S5. The MD studies of compound 9 binding to β2-AR (a) Protein-ligand RMSD; (b) Protein RMSF; (c) Interactions and contacts between β2-AR and compound 9 throughout MD.
