# Supplementary material for: Integration of Two-Dimensional Liquid Chromatography-Mass Spectrometry and Molecular Docking to Characterize and Predict Polar Active Compounds in Curcuma kwangsiensis
Source: Molecules. 2022 Nov 9;27(22):7715. doi: 10.3390/molecules27227715 (PMC9692749; doi:10.3390/molecules27227715)
Supplement: Supplementary file 1 [file molecules-27-07715-s001.zip › molecules-2003988-supplementary.pdf]

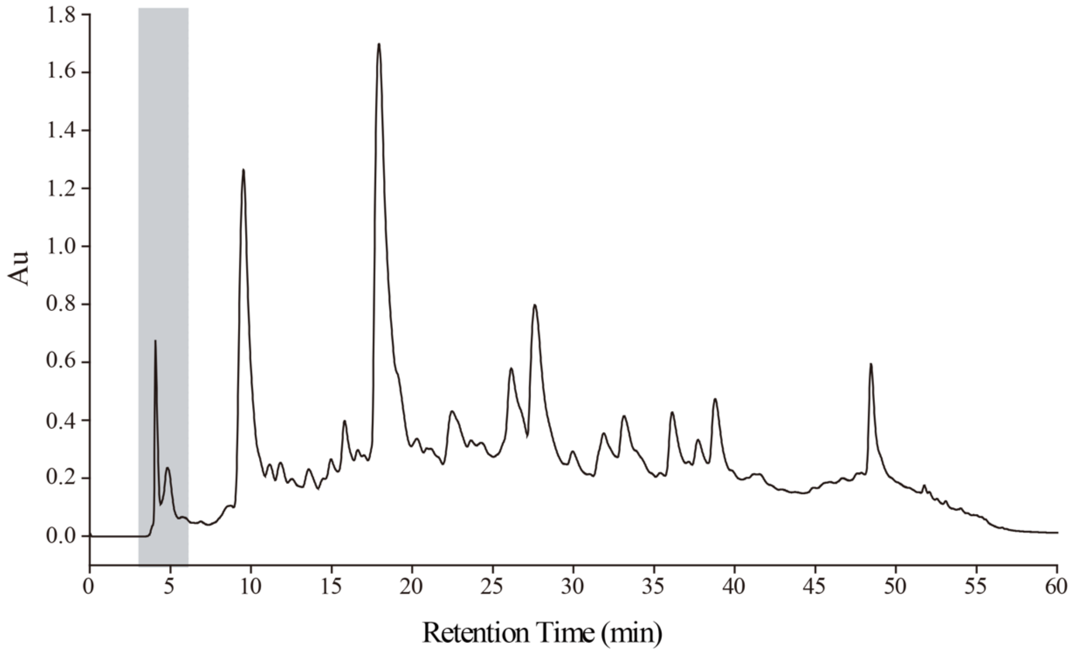

Figure S1 HPLC preparation of polar fraction on the Unitary C18 column (265 mm × 50 mm i.d., 7 μm)

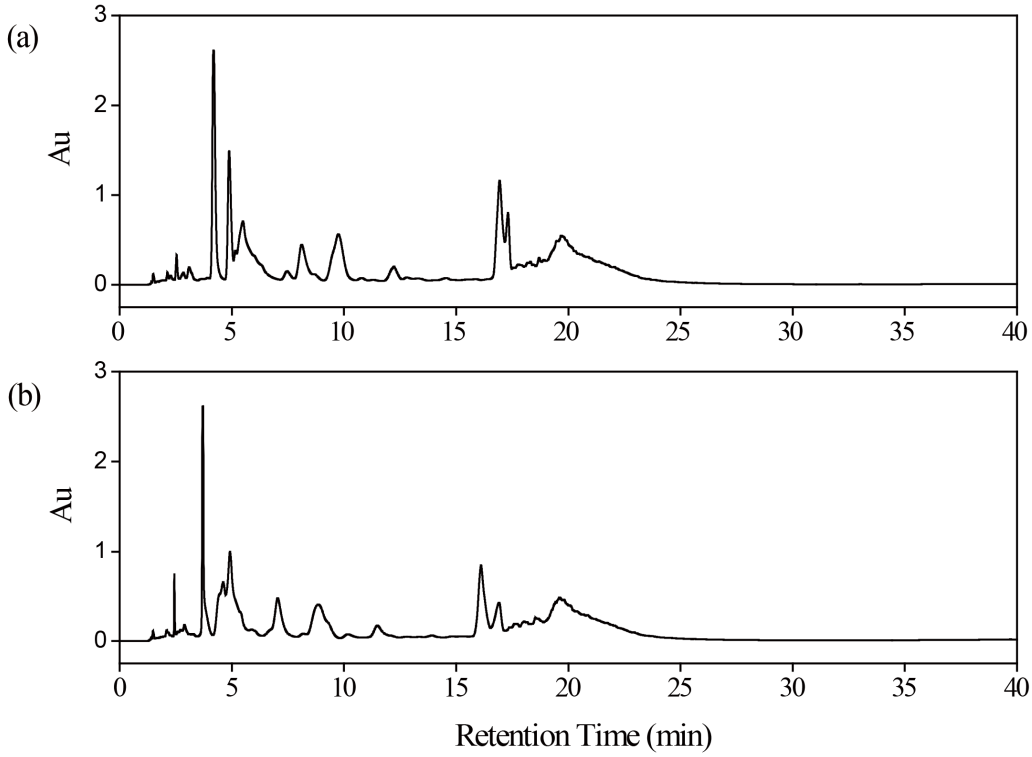

Figure S2 RPLC chromatograms of polar fraction at 254 nm under (a) mobile phase A: 0.1% formic acid in water (v/v), mobile phase B: 0.1% formic acid in MeOH (v/v), (b) mobile phase A: 0.1% formic acid in water (v/v), mobile phase B: 0.1% formic acid in ACN (v/v).

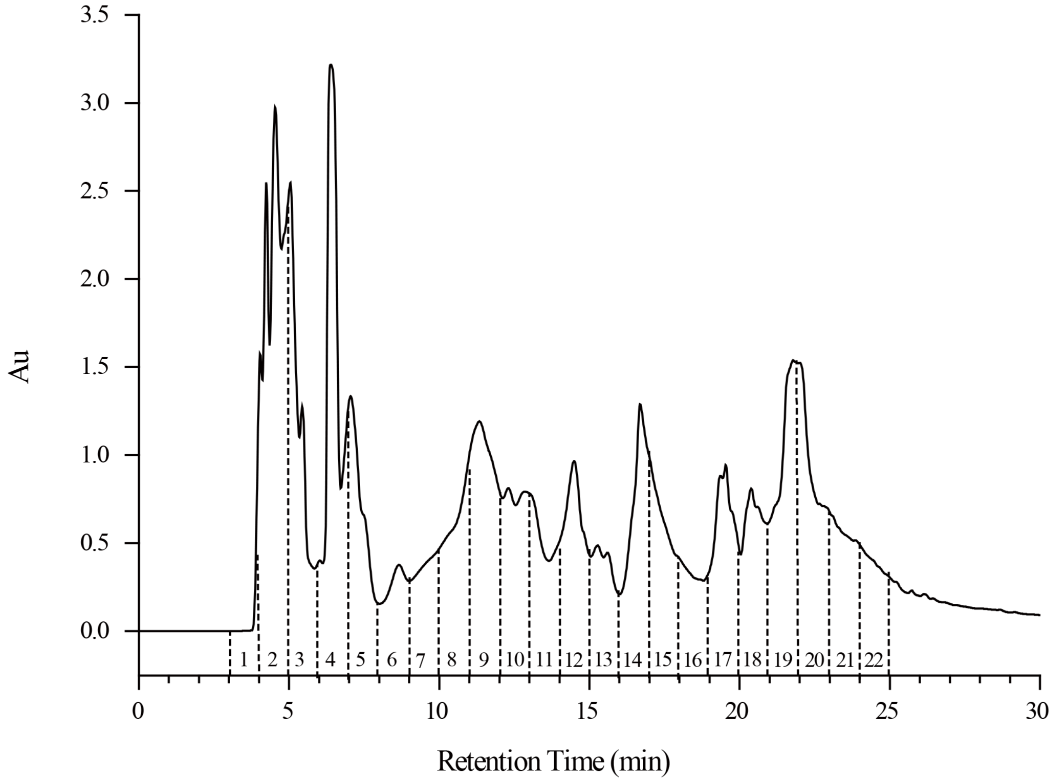

Figure S3 The first-dimension separation of the polar fraction at 254 nm on the XAqua C18 column (250 mm × 10 mm i.d., 5 μm).

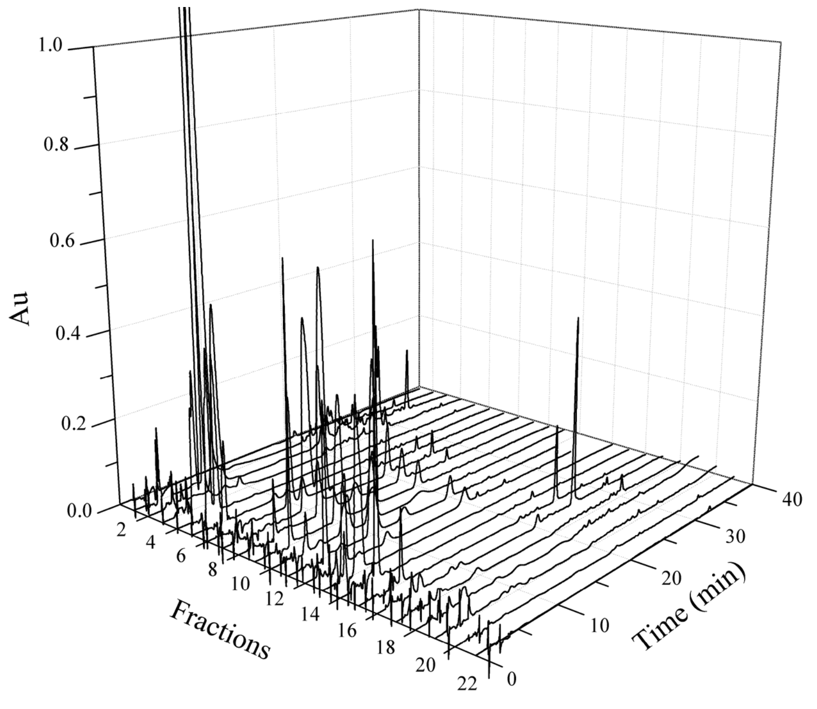

Figure S4 The three-dimensional chromatogram of 22 fractions at 254 nm analyzed on the XAmide column (150 × 2.1 mm i.d., 5 μm).

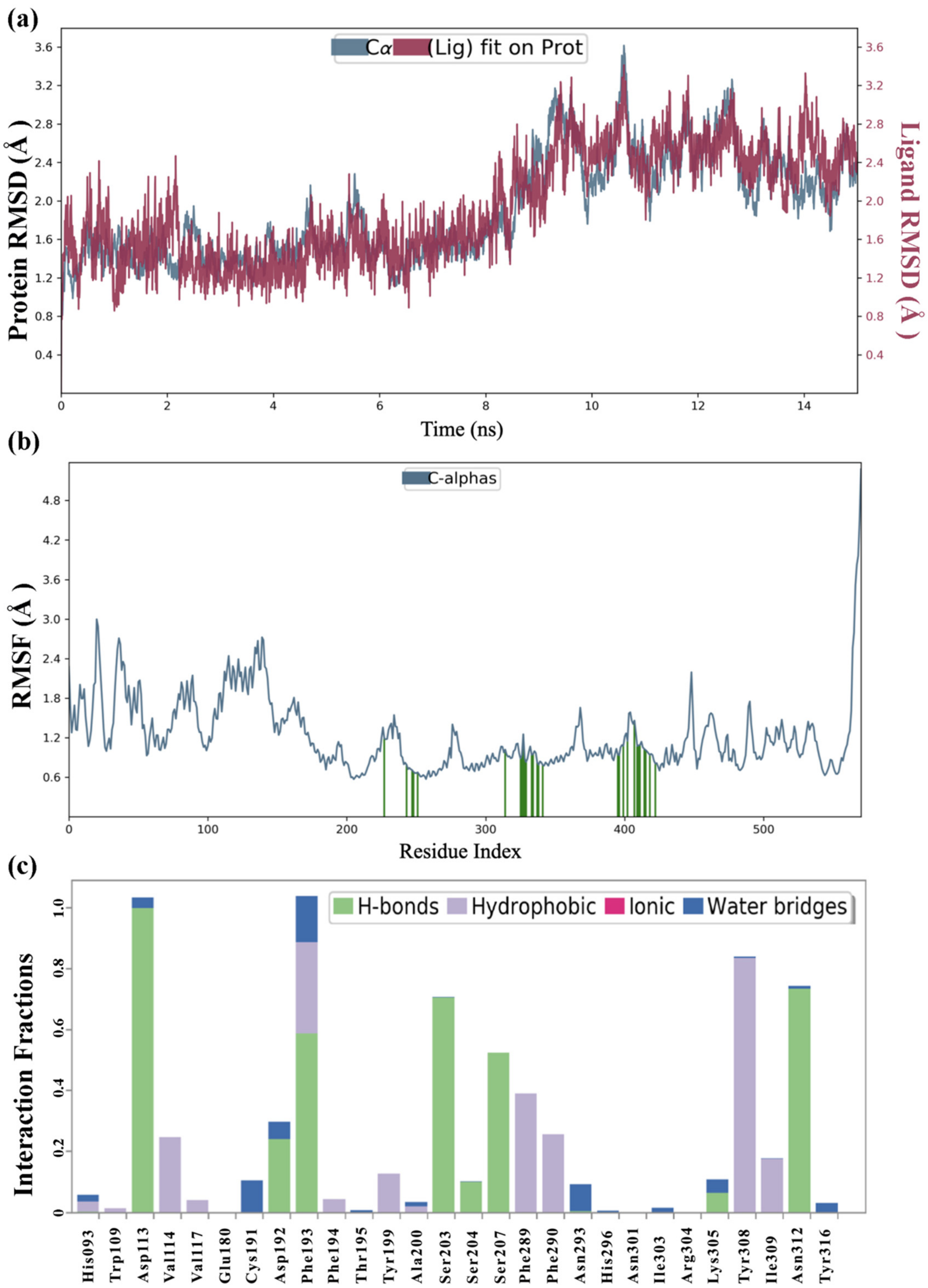

Figure S5 The MD studies of compound 9 binding to β2-AR (a) Protein-ligand RMSD (b) Protein RMSF. (c) Interactions and contacts between β2-AR and compound 9 throughout MD.
